# Tumor stiffness measured by shear wave elastography correlates with tumor hypoxia as well as histologic biomarkers in breast cancer

**DOI:** 10.1186/s40644-020-00362-7

**Published:** 2020-12-01

**Authors:** Joonghyun Yoo, Bo Kyoung Seo, Eun Kyung Park, Myoungae Kwon, Hoiseon Jeong, Kyu Ran Cho, Ok Hee Woo, Sung Eun Song, Jaehyung Cha

**Affiliations:** 1 Department of Radiology, Korea University Ansan Hospital, Korea University College of Medicine, 123 Jeokgeum-ro, Danwon-gu, Ansan-si, Gyeonggi-do 15355 South Korea; 2 Department of Pathology, Korea University Ansan Hospital, Korea University College of Medicine, 123 Jeokgeum-ro, Danwon-gu, Ansan-si, Gyeonggi-do 15355 South Korea; 3 Department of Radiology, Korea University Anam Hospital, Korea University College of Medicine, 73 Goryeodae-ro, Seongbuk-gu, Seoul, 02841 South Korea; 4 Department of Radiology, Korea University Guro Hospital, Korea University College of Medicine, 148 Gurodong-ro, Guro-gu, Seoul, 08308 South Korea; 5 Medical Science Research Center, Korea University Ansan Hospital, 123 Jeokgeum-ro, Danwon-gu, Ansan-si, Gyeonggi-do 15355 South Korea

**Keywords:** Ultrasonography, Elastography, Breast cancer, Hypoxia, Biomarkers, Tumor microenvironment

## Abstract

**Background:**

Shear wave elastography (SWE) is an ultrasound technique for the noninvasive quantification of tissue stiffness. The hypoxic tumor microenvironment promotes tumor stiffness and is associated with poor prognosis in cancer. We aimed to investigate the correlation between tumor hypoxia and histologic biomarkers and tumor stiffness measured by SWE in breast cancer.

**Methods:**

From June 2016 to January 2018, 82 women with invasive breast cancer who underwent SWE before treatment were enrolled. Average tumor elasticity (E_average_) and tumor-to-fat elasticity ratio (E_ratio_) were extracted from SWE. Immunohistochemical staining of glucose transporter 1 (GLUT1) was used to assess tumor hypoxia in breast cancer tissues and automated digital image analysis was performed to assess GLUT1 activities. Spearman correlation and logistic regression analyses were performed to identify associations between GLUT1 expression and SWE values, histologic biomarkers, and molecular subtypes. The Mann–Whitney *U* test, *t* test, or Kruskal–Wallis test was used to compare SWE values and histologic features according to the GLUT1 expression (≤the median vs > median).

**Results:**

E_average_ (*r* = 0.676) and E_ratio_ (*r* = 0.411) correlated significantly with GLUT1 expression (both *p* <  0.001). E_average_ was significantly higher in cancers with estrogen receptor (ER)–, progesterone receptor (PR)–, Ki67+, and high-grade (*p* <  0.05). E_ratio_ was higher in cancers with Ki67+, lymph node metastasis, and high-grade (*p* <  0.05). Cancers with high GLUT1 expression (>median) had higher E_average_ (mean, 85.4 kPa vs 125.5 kPa) and E_ratio_ (mean, 11.7 vs 17.9), and more frequent ER– (21.7% vs 78.3%), PR– (26.4% vs 73.1%), Ki67+ (31.7%% vs 68.3%), human epidermal growth factor receptor 2 (HER2) + (25.0% vs 75.0%), high-grade (28.6% vs 71.4%), and HER2-overexpressing (25.0% vs 75.0%) and triple-negative (23.1% vs 76.9%) subtypes (*p* <  0.05). Multivariable analysis showed that E_average_ was independently associated with GLUT1 expression (*p* <  0.001).

**Conclusions:**

Tumor stiffness on SWE is significantly correlated with tumor hypoxia as well as histologic biomarkers. In particular, E_average_ on SWE has independent prognostic significance for tumor hypoxia in the multivariable analysis and can potentially be used as a noninvasive imaging biomarker to predict prognosis and pretreatment risk stratification in breast cancer patients.

## Background

Tumor stiffness in breast cancer is an indicator of poor prognosis. Changes in extracellular matrix (ECM) and endothelium stiffness lead to increased interstitial pressure and reduced tumor perfusion and drug delivery. The resulting tumor stiffness can promote tumor invasion and metastasis [1, 2]. Shear wave elastography (SWE) is a highly reproducible ultrasound (US) technique for the noninvasive quantification of tissue stiffness [3]. In this method, an initial US push pulse, which induces a shear wave perpendicular to the US beam, is applied to the tissue [4]. The speed of the shear wave generated through the tissues is calculated, and the strain modulus is estimated in kilopascals (kPa) from the speed of sound. The elastic modulus of the tissue is proportional to the square of the shear wave speed (E = 3pc^2^; where E is elasticity, p is the density of the tissue, and c is the shear wave speed). Usually, breast cancer is harder than the surrounding normal breast tissue or fat. Shear waves pass faster through hard tissue than soft tissue, and cancer usually has a higher stiffness value when expressed in kPa. SWE has been reported to be a convenient and effective way to distinguish between benign and malignant breast masses without loss of sensitivity [5]. Several studies have reported that tumor stiffness values on SWE for breast cancer are associated with prognostic pathological indicators such as immunohistochemical profile, molecular subtypes, or lymphovascular invasion [6–12]. In addition, the multivariable analysis by Evans et al. [13] demonstrated that preoperative tumor stiffness on SWE was a significant independent prognostic indicator of breast cancer-specific survival. Therefore, identifying the primary histologic cause of tumor stiffness on SWE is important for the use of SWE in oncology imaging.

The hypoxic tumor microenvironment promotes tumor stiffness by remodeling the ECM and increasing collagen content and collagen crosslinks [2, 14, 15]. According to a recent study using a mouse tumor model, tumor stiffness on elastography is related to total fibrous collagen content and collagen crosslinks, which may be a possible way to assess changes in the tumor microenvironment, especially in ECM [16]. In breast cancer, hypoxia is very important because it is strongly associated with angiogenesis, cancer growth, metastasis, and resistance to treatment [17]. Hypoxia induces the secretion of matrix metalloproteinases, which causes invasion of cancer through ECM degradation, penetration of the walls of blood vessels and lymphatic vessels, and promotion of metastasis [17–19]. In addition, tumor stiffness induced by hypoxia affects the vascular endothelium through the CCN1–β-catenin–N-cadherin pathway, which promotes the binding of cancer cells to blood vessels and contributes to the metastatic cascade and promotion of metastasis [18]. If tumor stiffness measured by SWE is associated with hypoxic tumor microenvironment, the SWE parameters can be used as potential imaging biomarkers to predict prognosis and pretreatment risk stratification in breast cancer patients.

We conducted this study to investigate the relationships between quantitative stiffness parameters on SWE and tumor hypoxia and prognostic histologic biomarkers in invasive breast cancers. Immunohistochemical staining was performed to examine the expression of hypoxia-related endogenous protein, glucose transporter-1 protein (GLUT1) and digital image analysis was used for quantitative immunohistochemical evaluation [20–23]. GLUT1 is a high-affinity glucose transporter that regulates glucose uptake [22]. GLUT1 expression increases under hypoxia, which creases greater dependence on glycolysis as an energy source. Increased glucose consumption can provide the energy needed for tumor cell proliferation. Among the many immunohistochemical indicators of hypoxia, GLUT1 is overwhelmingly expressed in breast cancer but its expression is extremely rare in benign breast lesions such as ductal hyperplasia or atypical ductal hyperplasia. GLUT1 expression correlates significantly with survival outcomes and prognostic factors in breast cancer [20, 23, 24]. Therefore, we chose to assess GLUT1 expression because it is useful for measuring hypoxia in breast cancer and we assessed its association with prognostic factors. We evaluated tumor grade, lymph node, and estrogen receptor (ER), progesterone receptor (PR), human epidermal growth factor receptor 2 (HER2), and Ki67 as histologic biomarkers.

## Methods

### Patients

**Fig. 1:**
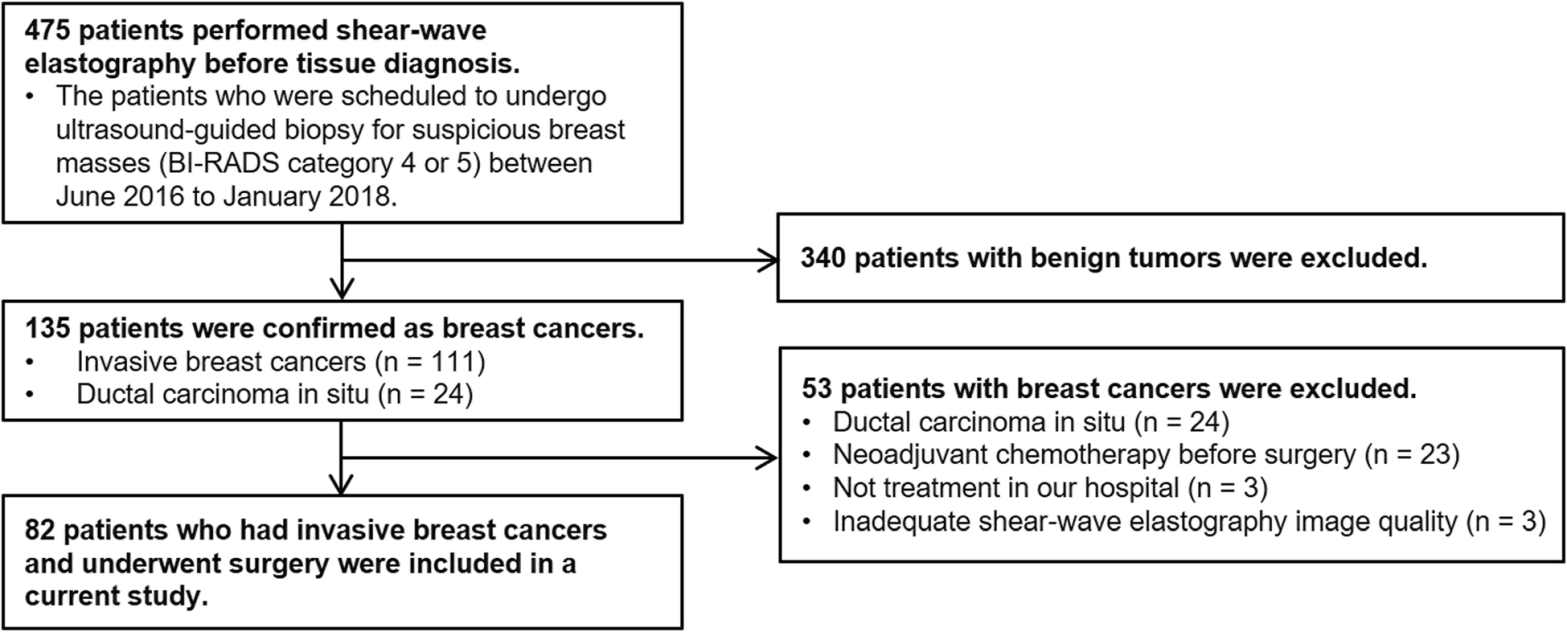
Flowchart of patients included

This retrospective study was approved by our institutional review board, which waived the requirement for informed consent. From June 2016 to January 2018, 475 consecutive women with suspicious breast masses, which were assessed as category 4 or 5 according to the Breast Imaging Reporting and Data System US lexicon [25], underwent B-mode US and SWE before US-guided tissue diagnosis. Of 475 patients, 82 patients who were identified with pathologically invasive breast cancer and underwent surgery without neoadjuvant chemotherapy were included in this study (Fig. 1). The mean age of the patients was 53.9 years (range, 36─85 years). Three hundred ninety-three patients were excluded for the following reasons: (a) the pathology diagnosis was benign tumor (*n* = 340) or ductal carcinoma in situ (*n* = 24), (b) neoadjuvant chemotherapy was performed before surgery (*n* = 23), (c) the patient was not treated in our hospital (*n* = 3), or (d) the SWE image quality was not suitable for evaluation (*n* = 3).

### SWE analysis

B-mode US and SWE images were obtained using an Aplio 500 US system (Canon Medical Systems, Tokyo, Japan) equipped with a 5–14 MHz linear-array transducer, by one of two radiologists (B.K.S and E.K.P. with 18 years and 8 years of experience in breast imaging, respectively). The radiologists were given information about the clinical history and mammography results at the time of the US examination. They performed whole-breast B-mode US and obtained at least two orthogonal images for each suspicious breast mass. After B-mode US, SWE was performed for the suspicious mass by the same radiologist who performed the B-mode US.

The mass was located in the center of the elasticity boxes on a plane showing the longest diameter, and SWE images were obtained without compression. The elasticity boxes included the mass and surrounding tissues. The US transducer was held over the mass for a few seconds to stabilize the SWE image before the image was saved for SWE measurements. The elasticity color map was overlaid on the B-mode image; the softest parts were displayed in blue and the hardest parts in red. Quantitative elasticity parameters were measured on the elasticity color map image using the US system’s built-in quantification tool. The default quantitative scale ranged from 0 to 200 kPa. Two round 2-mm-diameter regions of interests were positioned on the hardest part of the mass and adjacent adipose tissue.

**Fig. 2:**
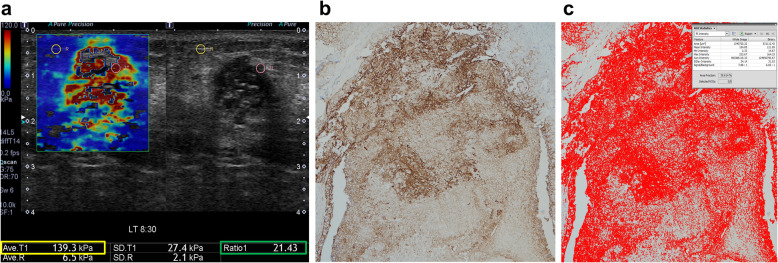
Quantitative measurement of tumor stiffness on shear-wave elastography (SWE) and immunohistochemical staining. **a**. Quantitative elasticity parameters were measured on the elasticity color map image. Two round 2-mm-diameter regions of interests were positioned on the hardest part of the mass and adjacent adipose tissue. The ultrasound system automatically displayed average elasticity of the tumor (E_average_) with standard deviation, average elasticity of the adjacent fat with standard deviation, and elasticity ratio between the tumor’s average elasticity and the average elasticity of adjacent fat (E_ratio_). Two quantitative SWE parameters, E_average_ (yellow box) and E_ratio_ (green box), were extracted to investigate the relationships between stiffness parameters on SWE and immunohistochemical staining. **b–c**. On immunohistochemical staining, the area fraction of positive reactions was measured. Each color image was converted to a binary image. The positive areas—brown area for glucose transporter-1 protein (GLUT1) staining (× 100) (**b**)—were converted to red (**c**). The area fraction of positive GLUT1 reaction was determined as the percentage of red pixels in the binary image

The US system automatically displayed the SWE parameters such as average elasticity of the tumor (E_average_) with standard deviation, average elasticity of the adjacent fat with standard deviation, and elasticity ratio between the tumor’s average elasticity and the average elasticity of adjacent fat (E_ratio_) (Fig. 2a). We extracted the two quantitative SWE parameters E_average_ and E_ratio_, and used these to examine the relationships between stiffness parameters on SWE and immunohistochemical staining for intratumoral hypoxia in breast cancers. In our hospital, we repeated the SWE examination three times for each suspicious breast mass and measured E_average_ and E_ratio_ values three times for each mass. We used the mean of the three measurements of E_average_ and E_ratio_ for statistical analysis.

### Histopathology analysis

Breast cancer tissues obtained from surgical specimens were stained immunohistochemically to evaluate the degree of tumor hypoxia using anti-GLUT1 antibody. All formalin-fixed paraffin-embedded tissues were sectioned at a thickness of 4 μm for staining. Sections were deparaffinized in xylene and rehydrated for 5 min per session in a graded series of 100, 95, 80, and 70% alcohol. Antigen retrieval was performed by heating in a pressure cooker for 15 min. To reduce nonspecific background staining, tissue sections were incubated in a hydrogen peroxide blocking agent. The slides were washed in Tris-buffered saline and incubated with the protein block (Novolink polymer detection kit; Leica Biosystems, Newcastle upon Tyne, UK). Primary rabbit anti-GLUT1 antibody (ab15309; Abcam, Cambridge, UK) was diluted 1:200 with goat serum. For GLUT1 staining, hypoxic areas stain brown.

Automated digital microscopic image analysis was used for the quantitative assessment of the degree of staining. A pathologist (H.J. with 14 years of experience) evaluated the slides under low magnification (× 40) and chose the most positively stained areas for each tumor. Subsequently, three nonoverlapping fields were selected under high magnification (× 100) for each slide. Images of these fields were captured with the camera and then imported to the digital image analysis software (Eclipse Ni and NIS-Elements BR; Nikon, Ofuna, Japan). The area fraction of positive reactions for immunohistochemistry was measured [26]. Each color image was converted to a binary image. The positive areas—brown area for GLUT1 staining—were converted to red (Fig. 2b–c). The area fraction of positive reaction was determined as the percentage of red pixels in the binary image. The area fraction was measured in three nonoverlapping fields on each slide, and the mean value of the area fraction was used for statistical analysis. The necrotic sites were not included in the evaluation.

We reviewed the histology reports for evaluation of prognostic biomarkers of breast cancer and dichotomized the results according to the tumor grade and the status of lymph node metastasis, ER, PR, HER2, and Ki67. Tumor grade was classified as 1, 2, or 3 using the Nottingham scoring system and then dichotomized as low (grades 1 and 2) and high (grade 3) [27]. For the ER and PR, the Allred scoring system was used, and a score > 2 was considered as positive [28]. HER2 overexpression was considered positive when membranes were graded 3+ or 2+ HER2 staining on immunohistochemistry with HER2 gene amplification in silver-stained in situ hybridization. The Ki67 index was defined as positive if the expression was > 20%. Based on the ER, PR, HER2, and Ki67 results, the cancers were divided into four molecular subtypes: luminal A cancer (ER and/or PR+, HER2–, and Ki67–); luminal B cancer (ER and/or PR+, HER2–, and Ki67+, or ER and/or PR+, HER2+, and Ki67±); HER2-overexpressing cancer (ER–, PR–, and HER2+), and triple-negative cancer (ER–, PR–, and HER2–) [29].

### Statistical analysis

Spearman’s rank correlation analysis was used to identify correlations between SWE values (E_average_ and E_ratio_) and the area fraction of positive reactions in GLUT1 staining. The Mann–Whitney *U* test (for data without a normal distribution) or t test (for data with a normal distribution) was used to identify correlations between SWE parameters and histologic biomarkers such as tumor grade and lymph node, ER, PR, HER2, and Ki67 status. SWE parameters were compared between the four molecular subtypes of breast cancer using the Kruskal–Wallis test and pairwise comparisons with the Bonferroni post hoc correction. To compare SWE values and the status of histologic biomarkers and molecular subtypes based on the area fractions of positive GLUT1 staining (≤ the median vs > median), the Mann–Whitney *U* test, *t* test, or Kruskal–Wallis test was used. Univariable and multivariable logistic regression analysis was performed to assess correlations between GLUT1 expression and SWE values and histologic features. Multivariable regression analysis was performed to identify variables that were independently associated with GLUT1 expression using the statistically significant variables in the univariable analysis. *P* values < 0.05 were considered to indicate significant differences. Statistical analysis was performed by a biostatistician (J.C.) using commercially available statistical software (SPSS, version 25.0; IBM Corp., Armonk, NY, USA).

## Results

The mean size of the breast cancer was 21.9 mm (range, 8–50 mm). The histological types of the 82 invasive breast cancers included invasive ductal carcinoma (*n* = 75, 91.5%), invasive lobular carcinoma (*n* = 3, 3.7%), invasive micropapillary carcinoma (*n* = 2, 2.4%), tubular carcinoma (*n* = 1, 1.2%), and mucinous carcinoma (*n* = 1, 1.2%) (Table 1).

**Table 1:** Patient characteristics

Characteristics	No. of Tumors (*n* = 82)
Histological type	
invasive ductal carcinoma	75 (91.5)
invasive lobular carcinoma	3 (3.7)
invasive micropapillary carcinoma	2 (2.4)
tubular carcinoma	1 (1.2)
mucinous carcinoma	1 1.2)
Lymph node status	
negative	46 (56.1)
positive	36 (43.9)
ER status	
negative	23 (28.0)
positive	59 (72.0)
PR status	
negative	26 (31.7)
positive	56 (68.3)
HER2 status	
negative	66 (80.5)
positive	16 (19.5)
Ki67 status	
negative	41 (50.0)
positive	41 (50.0)
Tumor grade	
1 or 2	54 (65.9)
3	28 (34.1)
Molecular subtype	
luminal A cancer	26 (31.7)
luminal B cancer	35 (42.7)
HER2-overexpressing cancer	8 (9.8)
triple-negative cancer	13 (15.8)

Data are value with percentages in parentheses

*ER* estrogen receptor, *PR* progesterone receptor, *HER2* human epidermal growth factor receptor 2

### Correlations between SWE values and tumor hypoxia and histologic biomarkers

In the 82 invasive breast cancers, the mean E_average_ was 105.5 ± 30.6 kPa (range, 23.5–150.5 kPa; median, 113.4 kPa) and the mean E_ratio_ was 14.8 ± 8.4 (range, 3.0–60.8; median, 13.9). The mean area fraction of positive reaction in GLUT1 staining was 35.4 ± 15.0% (range, 6.6–78.4%; median 32.9%). The Spearman’s rank correlation coefficients between SWE values and the area fractions of positive reaction in GLUT1 staining were 0.676 for E_average_ and 0.411 for E_ratio,_ which were moderate correlations [30]. The correlations between SWE values and GLUT1 staining were significant (*p* <  0.001 for all).

Table 2 shows the relationships between SWE values and histological biomarkers in the 82 invasive breast cancers. E_average_ values were significantly higher in breast cancers with ER negativity (*p* = 0.006), PR negativity (*p* = 0.009), Ki67 positivity (*p* = 0.002), and high grade (*p* = 0.002). However, E_average_ and E_ratio_ was not associated with lymph node metastasis or HER2 overexpression (both *p* > 0.05). E_ratio_ was significantly higher in breast cancers with lymph node positivity (*p* = 0.027), Ki67 positivity (*p* = 0.048), and high grade (*p* = 0.003). E_ratio_ values were not significantly associated with ER, PR, or HER2 status (all *p* > 0.05).

**Table 2:** Relationship between SWE values and histologic biomarkers of breast cancers

	E_average_	*p value	E_ratio_	*p value
Lymph node				
negative (*n* = 46)	108.1 (104.4 ± 31.6)	0.695	12.8 (12.8 ± 6.4)	0.027
positive (*n* = 36)	115.1 (106.8 ± 29.7)		16.2 (17.3 ± 10.1)	
ER				
negative (*n* = 23)	126.3 (118.5 ± 29.1)	0.006	15.9 (17.6 ± 11.2)	0.122
positive (*n* = 59)	109.0 (100.4 ± 29.9)		13.0 (13.7 ± 6.9)	
PR				
negative (*n* = 26)	124.8 (117.7 ± 27.9)	0.009	16.2 (17.6 ± 11.3)	0.134
positive (*n* = 56)	108.1 (99.8 ± 30.4)		13.1 (13.5 ± 6.5)	
HER2				
negative (*n* = 66)	111.6 (103.4 ± 31.7)	0.256	13.0 (13.6 ± 6.3)	0.085
positive (*n* = 16)	119.7 (114.1 ± 24.6)		15.6 (19.7 ± 13.5)	
Ki67				
negative (*n* = 41)	102.9 (95.0 ± 32.8)	0.002	12.8 (12.6 ± 5.8)	0.048
positive (*n* = 41)	119.2 (116.0 ± 24.5)		14.7 (17.0 ± 10.0)	
Tumor grade				
1 or 2 (*n* = 54)	104.4 (98.2 ± 30.9)	0.002	12.4 (13.3 ± 8.8)	0.003
3 (*n* = 28)	120.9 (119.5 ± 25.2)		17.2 (17.6 ± 7.0)	
Molecular subtype				
luminal A cancer (*n* = 26)	94.8 (91.4 ± 30.7)	0.009	12.4 (12.1 ± 5.5)	0.094
luminal B cancer (*n* = 35)	115.3 (108.2 ± 27.1)		14.2 (15.1 ± 7.5)	
HER2-overexpressing cancer (*n* = 8)	127.6 (118.0 ± 32.1)		22.4 (24.1 ± 16.5)	
triple-negative cancer (*n* = 13)	123.2 (118.5 ± 30.8)		13.2 (13.7 ± 4.9)	

Data are displayed as median (mean ± standard deviation)

*p values indicate comparisons between two groups using the Mann–Whitney *U* test or *t* test and four groups using the Kruskal–Wallis test

*ER* estrogen receptor, *PR* progesterone receptor, *HER2* human epidermal growth factor receptor 2

E_average_ differed significantly between the four molecular subtypes of breast cancer (*p* = 0.009). E_average_ was significantly higher in triple-negative cancers than in luminal A cancers when compared using the Kruskal–Wallis test and pairwise comparisons with Bonferroni correction (*p* = 0.030). E_average_ was higher in HER2-overexpressing cancers (*p* = 0.065) and luminal B cancers (*p* = 0.187) than in luminal A cancers, but these were not statistically significant. E_ratio_ did not differ between the molecular subtypes (*p* = 0.094).

### Factors influencing tumor hypoxia

The cancers were divided into two groups based on the median area fraction of GLUT1 staining (≤ the median vs > median). Lymph node metastasis, ER, PR, HER2, Ki67, and tumor grade status, and molecular subtypes were compared between the two groups (Table 3). Cancers with high GLUT1 expression had higher E_average_ (mean, 85.4 kPa vs 125.5 kPa, *p* <  0.001) and E_ratio_ (mean, 11.7 vs 17.9, *p* <  0.001) and higher rates of ER negativity (21.7% vs 78.3%, *p* = 0.001), PR negativity (26.4% vs 73.1%, *p* = 0.004), HER2 positivity (25.0% vs 75.0%, *p* = 0.026), Ki67 positivity (31.7%% vs 68.3%, *p* = 0.001), grade 3 cancer (28.6% vs 71.4%, *p* = 0.005), and HER2-overexpressing (25.0% vs 75.0%) and triple-negative (23.1% vs 76.9%) subtypes (*p* = 0.001).

**Table 3:** Differences in SWE values and histologic biomarkers according to GLUT1 expression

	GLUT1		*p value
	≤median (*n* = 41)	>median (*n* = 41)	
E_average_	85. 4 ± 29.3 kPa	125.5 ± 14.8 kPa	< 0.001
E_ratio_	11.7 ± 5.7	17.9 ± 9.6	< 0.001
Lymph node			0.373
negative	21 (45.7)	25 (54.3)	
positive	20 (55.6)	16 (44.4)	
ER			0.001
negative	5 (21.7)	18 (78.3)	
positive	36 (61.0)	23 (39.0)	
PR			0.004
negative	7 (26.9)	19 (73.1)	
positive	34 (60.7)	22 (39.3)	
HER2			0.026
negative	37 (56.1)	29 (43.9)	
positive	4 (25.0)	12 (75.0)	
Ki67			0.001
negative	28 (68.3)	13 (31.7)	
positive	13 (31.7)	28 (68.3)	
Tumor grade			0.005
1 or 2	33 (61.1)	21 (38.9)	
3	8 (28.6)	20 (71.4)	
Molecular subtype			0.001
luminal A cancer	21 (80.8)	5 (19.2)	
luminal B cancer	15 (42.9)	20 (57.1)	
HER2-overexpressing cancer	2 (25.0)	6 (75.0)	
triple-negative cancer	3 (23.1)	10 (76.9)	

Data refers to the numbers of subject included, means ± standard deviation for continuous variables and counts (%) for categorical variables

*p values indicate comparisons between two groups using the Mann–Whitney *U* test or *t* test and four groups using the Kruskal–Wallis test

*GLUT1* glucose transporter-1 protein, *ER* estrogen receptor, *PR* progesterone receptor, *HER2* human epidermal growth factor receptor 2

All variables except lymph node status were significantly associated with the area fraction of GLUT1 staining in the univariable regression analysis (Table 4). In the multivariable logistic regression analysis, E_average_ was strongly associated with GLUT1 expression (*p* < 0.001) but the remaining variables were not related to GLUT1 expression (*p*> 0.05).

**Table 4:** Univariable and multivariable logistic regression analysis of factors influencing GLUT1 expression

Variable		Univariable analysis			Multivariable analysis		
		β coefficient	Odd ratio (95% CI)	*p* value	β coefficient	Odd ratio (95% CI)	*p* value
E_average_		0.088	1.091 (1.049 − 1.135)	< 0.001	0.085	1.089 (1.041 − 1.139)	< 0.001
E_ratio_		0.137	1.147 (1.055 − 1.246)	0.001	0.063	1.065 (0.946 − 1.199)	0.299
Lymph node	negative vs positive	−0.397	0.672 (0.280 − 1.615)	0.374			
ER	negative vs positive	1.729	5.635 (1.838 − 17.278)	0.002	20.473	>1000	0.999
PR	negative vs positive	1.434	4.195 (1.514 − 11.623)	0.006	0.198	1.219 (0.095 − 15.709)	0.879
HER2	negative vs positive	1.342	3.828 (1.117 − 13.116)	0.033	1.983	7.262 (0.634 − 83.159)	0.111
Ki67	negative vs positive	−1.535	0.216 (0.085 − 0.546)	0.001	−0.038	0.962 (0.098 − 9.487)	0.974
Tumor grade	1 or 2 vs 3	1.368	3.929 (1.466 − 10.527)	0.007	−0.358	0.699 (0.116 − 4.226)	0.697
Molecular subtype				0.003			0.524
	luminal A vs luminal B cancer	1.723	5.600 (1.716 − 18.278)	0.004	0.872	2.392 (0.238 − 24.001)	0.459
	luminal A vs HER2-overexpressing cancer	2.534	12.600 (1.934 − 82.087)	0.008	−20.426	0.000	0.999
	luminal A vs triple-negative cancer	2.639	14.000 (2.778 − 70.557)	0.001	−17.689	0.000	1.000

*GLUT1* glucose transporter-1 protein, *ER* estrogen receptor, *PR* progesterone receptor; *HER2* human epidermal growth factor receptor 2, *CI* confidence interval

## Discussion

In this study, we found that SWE tumor stiffness was related to the degree of tumor hypoxia as well as the status of histologic biomarkers. The SWE parameters, E_average_ (*r* = 0.676) and E_ratio_ (*r* = 0.411) correlated significantly with GLUT1, a hypoxia-related endogenous protein (*p* < 0.001). SWE parameters were significantly associated with histologic biomarkers such as the status of the lymph nodes, ER, PR, Ki67, tumor grade, and molecular subtypes (all *p* < 0.05), all of which can affect tumor prognosis and treatment planning. When we divided the cancers into two groups based on the area fraction of GLUT1 staining (≤ the median vs > median), cancers with a higher GLUT1 expression had increased E_average_ and E_ratio_ on SWE and higher frequencies of aggressive histologic biomarkers. However, only E_average_ remained significantly related to GLUT1 expression in the multivariable logistic regression analysis (*p* < 0.001).

Our study have strengths. Our exploratory study is perhaps the first to report the tumor stiffness values of SWE to act as an indicator for tumor hypoxia, which can be easily incorporated in clinical practice to predict prognosis and risk stratification. Our study provides information showing that tumor hypoxia may be the root cause of tumor stiffness on SWE. The hypoxic tumor microenvironment is strongly associated with cancer proliferation, metastasis, and resistance to treatment [17]. Because breast cancer is heterogeneous, sometimes the characteristics of the entire cancer cannot be represented as a single molecular subtype in the pathology results, and the treatment response and prognosis may also differ within the same molecular subtype. Even if the molecular subtype has a pathologically good prognosis (e.g. luminal-A type), breast cancers with a high SWE value may be considered for aggressive treatments such as addition of anticancer drugs as well as routine antihormonal therapy. Our results provide information that may be helpful for improving patient outcomes by classifying patients into risk groups according to numerical categories based on the quantitative SWE values. This may improve the ability for predicting future prognosis at diagnosis, planning of optimal individualized treatments, and personalized monitoring.

Tumor cellularity, fibrosis, angiogenesis, or hypoxia are possible causes of tumor stiffness [2, 31–33]. In the present study, we focused on the tumor hypoxia. Because hypoxia stimulates angiogenesis and fibrogenesis, it can be a major factor of tumor stiffness and prognostic outcomes of breast cancer. Tumor hypoxia is common because of the inadequate oxygen delivery to areas of fast-growing cancer some distance from functional blood vessels. Previous studies have shown that hypoxia in breast cancer induces tissue stiffness through the involvement of lysyl oxidase, which promotes collagen crosslinking [2, 14, 15]. Lysyl oxidase is an extracellular amine oxidase that modifies collagens and elastin in the ECM by catalyzing the covalent crosslinking of fibers. Collagen crosslinking increases ECM tensile strength and focal adhesions. In breast cancer, increased expression of hypoxia-inducible factor 1 or hypoxia-induced lysyl oxidase family members increase collagen crosslinking and metastasis formation [14, 15]. In addition, tumor matrix stiffness affects the vascular endothelium through the CCN1–β-catenin–N-cadherin pathway and promotes metastasis [18]. Thus, hypoxia increases tumor stiffness by collagen crosslinking and fibrosis, and tumor stiffness promotes tumor progression and metastasis by changes in the vascular endothelium. After all, tumor hypoxia leads tumor hardness, progression, metastasis, and treatment resistance. Therefore, if we find a correlation between imaging characteristics and tumor hypoxia, imaging features can be used as a promising imaging biomarker to predict prognosis and treatment plans.

In this study, we analyzed the relationships between immunohistochemical GLUT1 staining and SWE tumor stiffness values (E_average_ and E_ratio),_ histologic biomarkers, and molecular subtypes. Cancer metabolism is characterized by high rates of glucose consumption. Hypoxia leads to upregulated glycolysis [24]. GLUT1 is the first member of the GLUT family and increased GLUT1 expression in cancer tissue reflects increased glycolytic metabolism. Thus, in breast cancer, GLUT1 expression indicates aggressive behavior and worse prognosis [20, 23, 24]. Previous studies have reported that GLUT1 is associated with high tumor grade, positive Ki67, negative hormone receptor, and triple-negative subtype in breast cancer [20, 23, 34]. Therefore, the GLUT1 expression level implies the degree of tumor proliferation and may be helpful guiding treatment planning. Our results are consistent with those of previous studies [20, 23, 24]. In this study, cancers with high GLUT1 expression had higher rates of ER negativity, PR negativity, HER2 positivity, Ki67 positivity, high-grade, and HER2-overexpressing and triple-negative subtypes. In addition, we found novel aspects in SWE such as that cancers with high stiffness values have significantly higher GLUT1 expression and that the average tumor elasticity (E_average_) of SWE was the only independent factor influencing tumor hypoxia in our multivariable logistic regression analysis. Our results suggest that the tumor stiffness on SWE in breast cancer may be an expression of tumor hypoxia, a primary poor prognostic indicator.

The correlations between SWE quantitative parameters and biomarkers of poor prognosis in breast cancer observed here are consistent with associations reported in previous studies [7–10]. In our study, the average tumor elasticity, expressed as E_average_, was significantly higher in breast cancers with ER negativity, PR negativity, Ki67 positivity, and high grade [9, 10]. The elasticity ratio between the average tumor elasticity and average elasticity of adjacent fat, expressed as the E_ratio_, was significantly higher in breast cancers with Ki67 positivity and high grade [8]. The correlations between SWE parameters and molecular subtypes in breast cancer observed here are consistent with associations shown in previous studies. The average elasticity of tumors is higher in triple-negative or HER2-overexpressing cancer compared with luminal type cancer [10]. In previous studies [13, 35–37], tumor stiffness on SWE was associated with survival outcomes and the response to neoadjuvant chemotherapy in breast cancer patients. Evans et al. [13] reported that the average tumor elasticity, tumor size on US, and ER status at the preoperative evaluation were independently associated with breast cancer-specific survival in a multivariable model. Tumor stiffness assessment before or during treatment was significantly associated with the response to neoadjuvant chemotherapy in breast cancer patients [35–37]. Production of collagen and increased collagen crosslinking in the ECM, of which collagen is a major component, in the hypoxic tumor microenvironment promote tumor stiffness, encourage tumor cell dissemination, and induce drug resistance [2, 14, 15, 18, 19, 38]. Thus, tumor stiffness affects the prognostic outcomes and drug response in cancer patients. Our study shows that SWE parameters are related to tumor hypoxia and histological prognostic biomarkers, and that the average tumor elasticity is the only significant independent factor influencing tumor hypoxia.

Our study had several limitations. First, the number of cancers was small. In our study, tumor stiffness parameters were measured three times on SWE for each lesion, and the means of the parameters were used for statistical analysis. Therefore, the measured SWE values were reliable for assessing correlations based on histopathological data. Second, the retrospective study design may have introduced a selection bias because only invasive breast cancers with preoperative SWE and surgical excision with accessible immunohistochemical data were included. Advanced cancers requiring neoadjuvant chemotherapy before surgery were excluded. Therefore, our results may not allow definite conclusions for all invasive breast cancers. Further studies with larger numbers of patients are needed. Third, we used two SWE stiffness parameters, E_average_ and E_ratio_. The US machine used in this study does not provide a minimum or maximum value for tumor stiffness, so only two quantitative values could be extracted. Fourth, in our routine clinical practice, one radiologist used SWE to assess the tumor stiffness of each breast tumor, and one of two experienced breast radiologists obtained the SWE images in this study, but we did not test the interobserver variability. However, SWE is known to be highly reproducible, and we used objective and quantitative SWE values for the analysis [3].

## Conclusions

SWE tumor stiffness values for invasive breast cancer are related mainly to the degree of tumor hypoxia. Hard cancers have significantly higher GLUT1 expression, an indicator of hypoxia, and levels of prognostic biomarkers such as ER negativity, PR negativity, Ki67 positivity, high grade, and HER2-overexpressing and triple-negative molecular subtypes. In particular, E_average_ of SWE had independent prognostic significance for tumor hypoxia in our multivariable analysis. The hypoxic tumor microenvironment in breast cancer is associated with cancer growth, metastasis, and resistance to treatment, all of which can lead to a poor prognosis. Therefore, quantitative information from SWE may be helpful for identifying the underlying tumor hypoxic microenvironment and histologic biomarkers, and for effective treatment planning and risk stratification.

## Data Availability

Please contact authors for data requests.

## Abbreviations

ECM: Extracellular matrix
SWE: Shear wave elastography
US: Ultrasound
kPa: Kilopascals
GLUT1: Glucose transporter-1 protein
ER: Estrogen receptor
PR: Progesterone receptor
HER2: Human epidermal growth factor receptor 2
E_average_: Average elasticity of the tumor
E_ratio_: Elasticity ratio between the tumor’s average elasticity and the average elasticity of adjacent fat
